# Muscle‐Specific Upregulation of *Timeless* Mediates Exercise‐Induced Amelioration of Age‐Related Circadian Rhythm Disruption and Cardiac Dysfunction in *Drosophila*

**DOI:** 10.1111/acel.70708

**Published:** 2026-09-07

**Authors:** Deng‐tai Wen, Shouzhi Lv, Jing‐yao Sun, Ying‐qi Chen, Ying Lin, Zhong‐rui Du, Gang‐bin Sun, Tian‐shuo Yuan, Deyu Shu, Wen‐qi Hou

**Affiliations:** ^1^ Ludong University Yantai Shandong Province China; ^2^ Anhui Institute of Information Technology Wuhu Anhui Province China

**Keywords:** aging, circadian rhythm, exercise, mitochondrial, oxidative stress, *Timeless*

## Abstract

The core genes of the circadian pathway, such as *Timeless(Tim)*, not only participate in the regulation of biological rhythms, but also play significant roles in DNA damage repair, chronic inflammation, and cellular metabolism. However, it remains unclear whether exercise can delay the age‐related phenotypic degeneration by regulating the muscle *Tim* gene. In here, we first carried out the expression regulation of the muscle *Tim* gene in the *Drosophila* by constructing the *Mhc‐gal4/Tim‐UAS* system, and then subjected the flies to a 4‐week endurance exercise intervention. The results showed that the knockdown of the muscle *Tim* gene accelerated aging‐related phenotypic deterioration in *Drosophila*, manifesting as increased nighttime activity, decreased climbing speed, elevated heart rate and reduced stroke volume, shortened time to hypoxic heart failure, and shortened lifespan. This is accompanied by reductions in muscle tissue levels of the *Clk* gene, *Sir2* gene, *PGC‐1α gene*, *Mhc* gene, MRCC‐I protein, and SOD protein, along with a significant increase in ROS. Conversely, overexpression of the muscle *Tim* gene delayed aging‐related phenotypic changes in aged *Drosophila*. Exercise not only effectively counteracts the acceleration of aging‐related phenotypic deterioration caused by muscle *Tim* gene knockdown but also further delayed aging‐related phenotypic changes in aged *Drosophila* on the basis of muscle *Tim* gene overexpression. In summary, this study highlights the role of the muscle *Tim* gene in the aging of skeletal muscles and the heart, as well as the relationship between exercise and the muscle *Tim* gene, providing strategies for the prevention and treatment of age‐related diseases.

## Graphical abstract

The muscle *Timeless* gene critically influences skeletal muscle, heart, and circadian rhythm aging by regulating the Timeless/Clock and Timeless/Sir2/PGC‐1α pathways, with exercise acting as an upstream modulator.

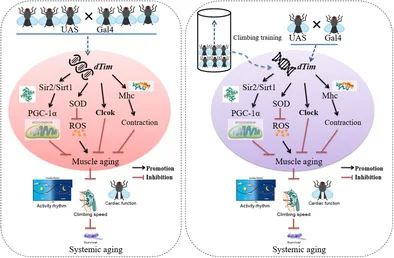

## 1 Introduction

Regardless of China or the entire world, population aging is becoming increasingly severe. According to UN data, in 2023, the global population aged 65 and over was approximately 800 million. It is projected that the global elderly population will exceed 1 billion for the first time in the 2030s. Global aging is accelerating, with currently about 10% of the world's population being over 65 (Mafra et al. 2025). In China, the population aged 60 and over is 310.31 million (approximately 310 million), accounting for 22.0% of the national population. China has already entered the stage of moderate aging and is progressing toward deep aging (Zhang, Yu, et al. 2025). With aging, both animals and humans experience a decline in tissue and organ function, as well as the occurrence of age‐related diseases such as sarcopenia, insomnia, and coronary heart disease (Hu et al. 2021). These diseases not only severely reduce the quality of life of the elderly but also impose a heavy economic burden on families and society (Cao et al. 2025). Therefore, it is particularly important for humanity to continuously delve into the molecular mechanisms of aging and then implement precise interventions to delay aging and reduce the incidence of age‐related diseases.

The *Tim* gene is a crucial regulatory gene in organisms, highly conserved from flies to humans. It plays a dual role in organisms, primarily acting as a regulator of the core circadian clock and a maintainer of DNA damage repair. In model organisms such as *Drosophila*, the core of the circadian clock consists of molecular feedback loops. The CLK/CYC heterodimer activates the transcription of *Per* and *Tim* genes, while the PER and TIM repressor proteins accumulate in the cytoplasm. Upon entering the nucleus, PER and TIM inhibit transcription by directly binding to the CLK/CYC heterodimer. This repression persists until the PER and TIM proteins are degraded, thereby allowing a new round of transcription. Light exposure causes rapid degradation of the TIM protein, resetting the circadian clock and enabling the organism to adapt to the day‐night cycle (Rakshit et al. 2012; Li et al. 2016). In mammals (including humans), although the *Tim* homolog is also involved in circadian clock regulation, its core role has shifted during evolution, becoming more focused on the cell cycle and DNA repair, while its circadian function is partially undertaken by other genes (such as *Cry* genes). When encountering DNA damage or replication stress, TIM stabilizes the replication fork, preventing its collapse (Mazzoccoli et al. 2016; Zhou et al. 2020). Furthermore, TIM interacts with proteins such as Tipin and participates in activating the S‐phase checkpoint. This means that if DNA is damaged, TIM helps send signals to pause the cell cycle, providing the cell with sufficient time to repair the damaged DNA, thereby preventing the transmission of mutations to daughter cells (Holzer et al. 2017; Leman et al. 2010). Consequently, by regulating the circadian clock, TIM indirectly influences the expression of downstream metabolism‐related genes, thereby affecting glucose metabolism and energy balance (Chen et al. 2024). However, the role of the muscle *Tim* gene in aging and aging‐related diseases remains unclear.

Numerous studies have confirmed that aerobic exercise is an effective means to delay aging and mitigate many aging‐related diseases, including coronary heart disease, Alzheimer's disease, circadian rhythm disorders, and sarcopenia (Li, Zhai, et al. 2024; Wen et al. 2020; Lefferts et al. 2022; Cools et al. 2025). However, the interactive relationship between exercise and the muscle *Tim* gene in the context of aging and aging‐related diseases remains unclear. This study leverages the advantages of *Drosophila* transgenic technology, their relatively short lifespan, and the conservation of exercise‐related mechanisms. By constructing an *Mhc/UAS‐Tim* system to achieve specific regulation (upregulation, normal expression, and downregulation) of Tim gene expression in *Drosophila* muscles, we will employ classic gain‐of‐function and loss‐of‐function approaches to investigate the effects of the muscle *Tim* gene on aging‐related circadian rhythms, exercise capacity, cardiac function, and the time to heart failure under hypoxia. Furthermore, we will analyze the underlying molecular mechanisms from the perspectives of muscle mitochondria, oxidative stress, and contractile proteins. Subsequently, we will verify the effects of exercise intervention under different conditions of muscle *Tim* expression, aiming to provide new strategies for delaying aging and preventing aging‐related diseases.

## 2 Materials and Methods

### 2.1 Fly Stocks, Husbandry, Grouping, Diet, and Exercise Training Protocols

The *Tim*‐UAS‐overexpression (*Tim*‐UAS‐OE) flies (stock ID: 80686; FlyBase Genotype: w*; P{UAS‐*Tim*.Y}2–1/CyO), and the Mhc‐gal4 (stock ID: 55133; FlyBase Genotype: w*; P{Mhc‐GAL4.K}2/TM3, Sb1) flies were obtained from the Bloomington Stock Center. The *Tim*‐UAS‐RNAi flies (stock ID: v2885; FlyBase Genotype: w^1118^; P{GD1267}v2885) were obtained from the Vienna Drosophila Resource Center. Female Mhc‐gal4 flies were crossed with male *Tim‐UAS‐OE* and *Tim*‐UAS‐RNAi flies. The F1 generation male offspring from these crosses were collected, and age‐matched male flies were divided into the following groups: *Tim*‐UAS‐OE>Mhc‐gal4(*OE*), *Tim*‐UAS‐OE>Mhc‐gal4 + Exercise (*OE*‐E), *Tim*‐UAS‐RNAi>Mhc‐gal4 (*RNAi*), and *Tim*‐UAS‐RNAi>Mhc‐gal4 + Exercise (*RNAi‐E*). Additionally, age‐matched male *Tim‐UAS‐OE(UAS)*, *Tim‐UAS‐OE* + Exercise *(UAS‐E)*, *Tim‐UAS‐RNAi(UAS)*, *Tim‐UAS‐RNAi* + Exercise (UAS‐E), and *Mhc‐gal4 (Gal4)* flies were used as control groups to account for genetic background.

All *Drosophila* were maintained and experimentally manipulated under controlled conditions (25°C, 50% humidity), with food replacement performed every other day. The food consisted of 2.0% yeast, 6.7% cornmeal, 0.7% agar, 1.6% soybean powder, 4.8% sucrose, 4.8% maltose, and 0.3% propionic acid (Wen et al. 2020).

Flies were exercised in vials with an 8‐cm length. The vials were rotated at 60 rad/s. After each up‐and‐down turn, the vials were held stationary for 10 s to allow the flies to climb. Flies were exercised for 60 or 65 min per day (Table 1). The exercise regimen consisted of 2 days of training followed by 1 day off, and then 2 days of training followed by 2 days off. All exercise groups began training at 2 weeks of age and underwent a 4‐week‐long exercise program (Mendez et al. 2016).

**TABLE 1:** Training protocols.

Day	Exercise	Rest	Exercise	Rest	Exercise	Rest	Exercise	Rest
Monday	15 min	5 min	15 min	5 min	15 min	5 min	15 min	5 min
Tuesday	20 min	5 min	15 min	5 min	15 min	5 min	15 min	5 min
Wednesday	Rest							
Thursday	15 min	5 min	15 min	5 min	15 min	5 min	15 min	5 min
Friday	20 min	5 min	20 min	5 min	15 min	5 min	15 min	5 min
Saturday	Rest							
Sunday	Rest							

### 2.2 Circadian Rhythm Measurement

**FIGURE 1:**
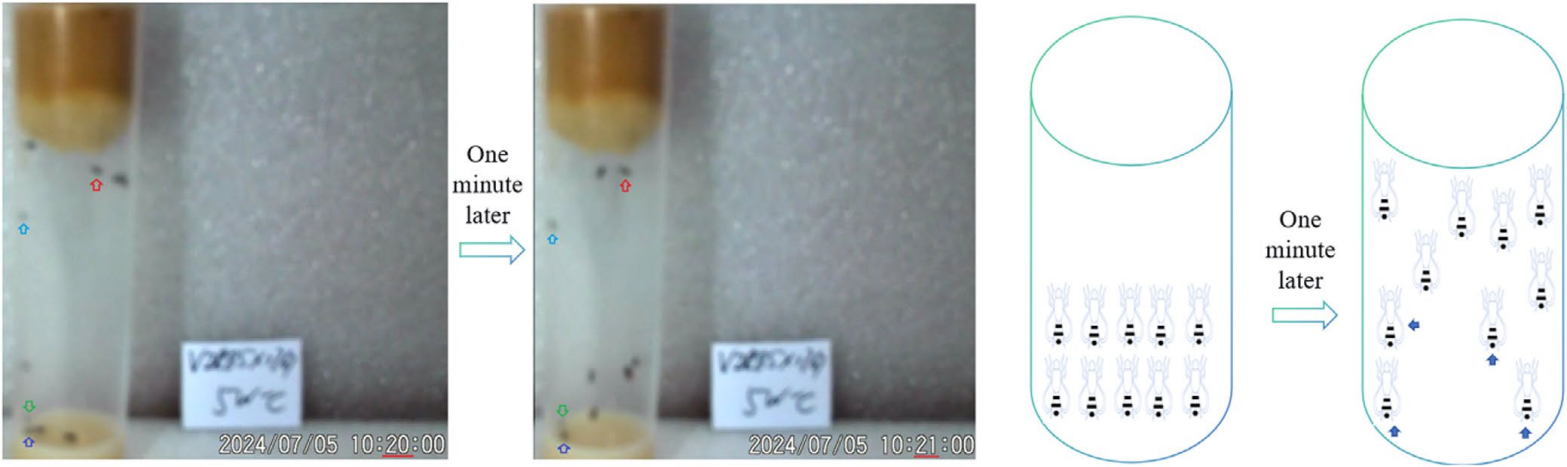
Method for analyzing locomotor activity rhythm of *Drosophila*. A 24‐h video recording was conducted on fruit flies in culture tubes. During analysis, the positions of the fruit flies in the video were analyzed at one‐minute intervals. If the position remained unchanged, it was scored as “0”; if the position changed, it was scored as “1.” The average activity rate per tube was then calculated (number of fruit flies with position changes divided by total number of fruit flies). As shown in the figure, during the time period from 10:20 to 10:21, six fruit flies exhibited position changes, resulting in an average activity rate of 0.4.

The circadian rhythm of the flies was measured at the end of 1 and 5 weeks of age. Ten flies from each group were placed in a culture tube and recorded for 24 h using a digital camera (infrared camera mode at night, Sony, 56 mega pixels). The video was analyzed using QQ video software (version: 4.6.3.1104). Each fly in the video was labeled “1” if it changed position within a 1‐min interval and “0” if it remained stationary (Figure 1). Using these labels, the following parameters were calculated: average activity rate per minute, average activity rate per hour (Average activity rate with 60 min), average activity rate during 12 h of daytime, Average activity rate during 12 h of night, and average activity rate over 24 h, and the 24‐h average activity rate curve of flies. These metrics provided a comprehensive assessment of the flies' circadian activity patterns.

### 2.3 Climbing Ability, Cardiac Function and Heart Failure, and Lifespan Assay

The climbing speed of *Drosophila* was used to reflect their motor performance. Specifically, the measurement procedure was as follows: the test vials containing flies were placed on a Power Tower platform, and the Power Tower mechanically tapped the vials every 10 s to knock the flies to the bottom (Tinkerhess et al. 2012). A video camera was then activated to record three consecutive climbing trials. The recordings were subsequently analyzed by AVS software, and screenshots were taken to capture the climbing height achieved within 3 s after each mechanical tap. The highest climbing height from each vial was recorded for analysis. Each group consisted of 20 flies, and the test was repeated five times per group (Yin et al. 2025).

Cardiac function assay: after the fruit flies were anesthetized in ether for 3 min, the head, ventral thorax, and ventral abdominal cuticle of anesthetized flies were removed, and the hearts were exposed. Oxygenated artificial hemolymph could maintain the normal function of the heart in *Drosophila*. High‐speed cameras captured video of a fruit fly's heart beating, and the video was recorded for 30 s. The video of heart rate and stroke volume was analyzed using AVS software (Ocorr et al. 2014). Stroke volume = (Square of dilation radius − Square of contraction radius) × π × 1 μm.

Heart failure assay: after the fruit flies completed the video analysis of their heart functions, the hemolymph from the fruit flies was removed using a pipette, and 5 μL of new hemolymph was then re‐introduced into the abdomen of the fruit flies. The time it took for the fruit fly's heart to go from normal beating to stopping beating was observed every 5 min. The time of fruit fly heart failure was analyzed by plotting the curve based on the duration of heart stoppage.

Dead flies were recorded daily to monitor mortality. The lifespan of each fly was calculated as the number of days from eclosion (emergence as an adult) until death. Survival curves were generated to characterize the lifespan distribution for each group. The sample sizes ranged from 200 to 210 flies per group to ensure robust statistical analysis (He and Jasper 2014).

### 2.4 Transmission Electron Microscopy of Skeletal Muscle

For electron microscopic observation, muscle tissues were meticulously dissected in an ice‐cold fixative solution containing 2.5% glutaraldehyde in 0.1 mol/L PIPES buffer (pH 7.4). After fixation at 4°C for 10 h, the samples were rinsed with 0.1 mol/L PIPES buffer. Post‐fixation was then carried out with 1% OsO_4_ for 30 min, followed by staining with 2% uranyl acetate for 1 h. Dehydration was performed through a graded ethanol series (50%, 70%, and 100%), after which the samples were embedded in epoxy resin. Ultrathin sections were prepared and examined using an HT‐7700 transmission electron microscope, and images were acquired for subsequent analysis (Lin et al. 2026).

### 2.5 ELISA Assay

The levels of MRCC‐I, SOD, and ROS were quantified using commercial ELISA kits (Insect MRCC‐I, SOD ELISA Kits, and ROS Kits, MLBIO, Shanghai, China). Muscles from 30 flies were homogenized in PBS (pH 7.2–7.4). The homogenates were snap‐frozen in liquid nitrogen and stored at 2°C–8°C after thawing. Samples were further homogenized using mechanical grinders and centrifuged at 2000–3000 rpm for 20 min, after which the supernatant was collected. For the assay, absorbance was measured at 450 nm within 15 min after adding the Stop Solution, using the blank well as the zero reference (Yin et al. 2025).

### 2.6 qRT‐PCR

At the end of the 5th week, skeletal muscle (thorax) samples from 30 *Drosophila* were collected per group and immersed in 1000 μL of Trizol reagent for subsequent analysis of relevant pathway gene expression (sample collection was repeated three times per group for experimental replicates). Each cDNA sample was prepared in triplicate for PCR amplification. Quantitative analysis was performed using the CT method (Lin et al. 2026). The primer sequences of *PGC‐1α* were as follows: F: 5′‐TGTTGCTGCTACTGCTGCTT‐3′; R: 5′‐GCCTCTGCATCACCTACACA‐3′. Primer sequences of *Tim* were as follows: F: 5′‐TAACACGAAGCCACGGAATAAA‐3′, R: 5′‐CTCGATGGTGTTCTCGGTGA‐3′. Primer sequences of *Clk* were as follows: F: 5′‐TCGGATTCAACGTCCATGTC‐3′, R: 5′‐GCTGTAACCCTTGAGGAGGAAAT‐3′. Primer sequences of *Mhc* were as follows: F: 5′‐3′:GACCGTGCGTAACGATAACTCC, R: 5′‐3′:GACTGCTGGGAGATGACACGA; Primer sequences of *Sir2* were as follows: F: 5′‐CAGCTCCTGTGGTATCCTGA‐3′; R: 5′‐GTGGAGAT GCTGTTGCTGTC‐3′. Primer sequences of *Rp49* were as follows: F: 5‐CTAAGCTGTCGCACAAATGG‐3′, R: 5′‐AACTTCTTGAATCCGGTGGG‐3′.

### 2.7 Statistical Analyses

The differences in various indicators among the Gal4 group, UAS group, and RNAi group or OE group were compared using one‐way ANOVA. The differences in various indicators between 1‐week‐old and 5‐week‐old *Drosophila*, as well as between the exercise and non‐exercise groups, were analyzed using an independent samples *t*‐test. The *p*‐values for the survival curves and heart failure time curves were determined using the log‐rank test. Experimental data are presented as mean ± standard deviation (SD), with statistical significance defined as *α* = 0.05 (or 0.01 where specified).

## 3 Results

### 3.1 The Muscle *Tim* Gene Is Involved in Regulating Age‐Related Phenotypic Changes in *Drosophila*

From fruit flies to humans, aging‐related phenotypic changes are highly conserved, manifested as decreased locomotor capacity, disrupted circadian rhythms, and weakened cardiac function (Chu et al. 2025; Li, Tan, and Zhao 2024; Chen et al. 2025). Among these, aging‐related lifespan is the most direct and ultimate phenotype (Kornadt et al. 2020). Both genetic predisposition and environmental factors can lead to differential susceptibility to aging among individuals, resulting in healthy aging versus pathological aging, and even the emergence of premature aging phenotypes. For instance, mutations in the *Sirt1* gene or long‐term high‐fat dietary intake can induce premature aging phenotypes (Wang et al. 2022; Yi et al. 2024). Alterations in circadian rhythms are among the earliest emerging phenotypes of aging and are closely associated with abnormal changes in the neuronal circadian pathway (Chandwaney et al. 1998). However, it remains unclear whether the circadian pathway in muscle participates in the regulation of the aging process.

**FIGURE 2:**
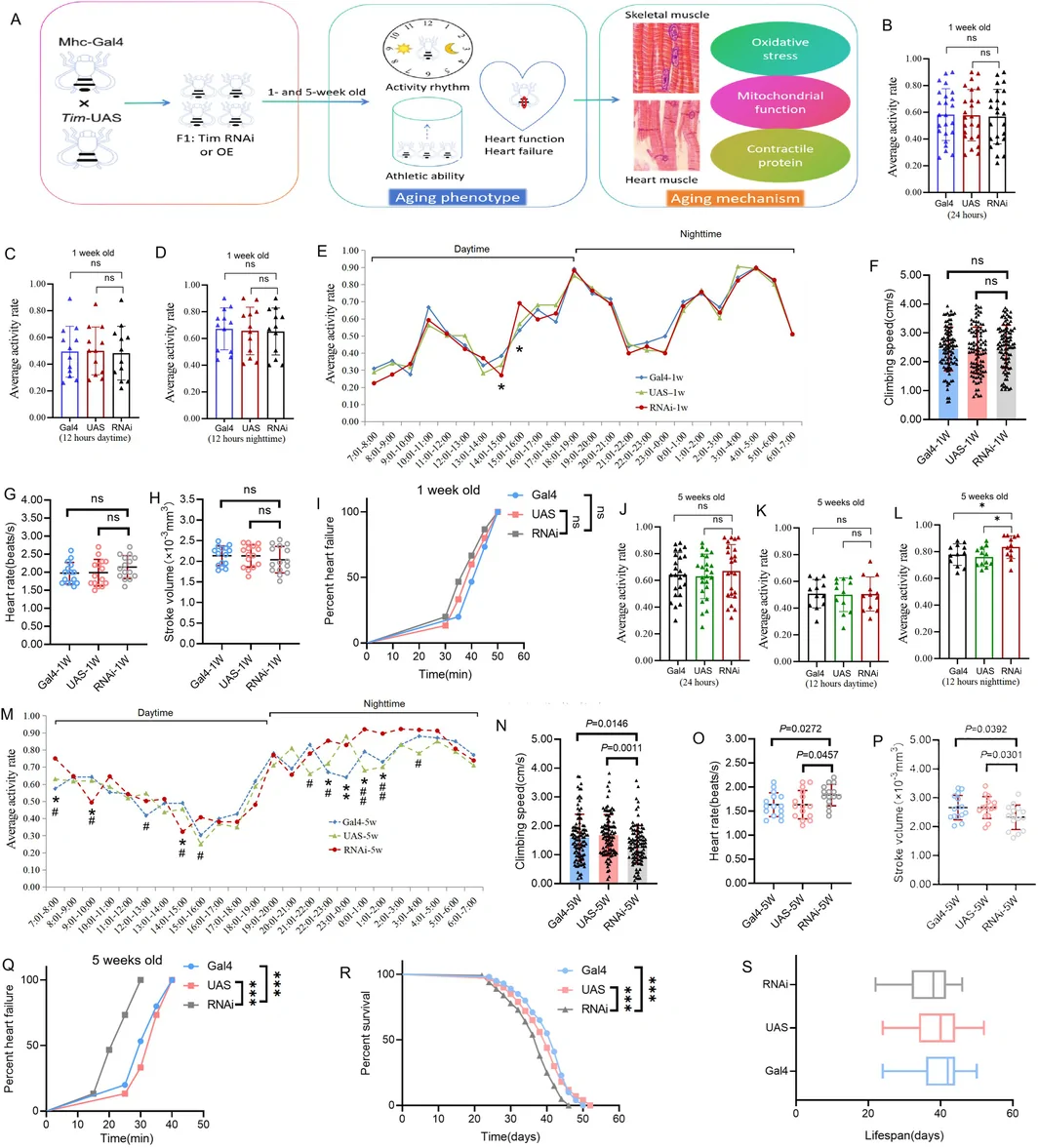
Effects of muscle‐specific *Tim* knockdown on aging‐related phenotypes in *Drosophila*. (A) Generation of *Drosophila* UAS/Mhc‐GAL4 system through genetic cross. (B) Average 24‐h locomotor activity of 1‐week‐old flies. (C) Average 12‐h daytime locomotor activity of 1‐week‐old flies. (D) Average 12‐h nighttime locomotor activity of 1‐week‐old flies. (E) Locomotor activity profile over 24 h of 1‐week‐old flies. (F) Climbing speed of 1‐week‐old flies. (G) Heart rate of 1‐week‐old flies. (H) Stroke volume of 1‐week‐old flies. (I) Time to hypoxic heart failure in 1‐week‐old flies. (J) Average 24‐h locomotor activity of 5‐week‐old flies. (K) Average 12‐h daytime locomotor activity of 5‐week‐old flies. (L) Average 12‐h nighttime locomotor activity of 5‐week‐old flies. (M) Locomotor activity profile over 24 h of 5‐week‐old flies. The locomotor activity of 5‐week‐old flies was significantly higher than that of UAS and GAL4 flies during the period from 20:01 to 04:00, suggesting that this may cause severe fragmentation of nighttime sleep in aged flies. (N) Climbing speed of 5‐week‐old flies. (O) Heart rate of 5‐week‐old flies. (P) Stroke volume of 5‐week‐old flies. (Q) Time to hypoxic heart failure in 5‐week‐old flies. (R) Survival curve of flies. (S) Average lifespan of flies. For comparisons among multiple groups (Gal4, UAS, and RNAi), one‐way analysis of variance (ANOVA) followed by least significant difference (LSD) post hoc tests was conducted. The log‐rank test was used to calculate *p*‐values for lifespan curve and time to hypoxic heart failure comparisons. Data are presented as means ± standard error of the mean (SEM). *p < 0.05; **p < 0.01; ns means no significant difference.

In this study, we established a *Tim‐UAS‐RNAi/Mhc‐Gal4* system in F1‐generation *Drosophila* through genetic crosses to achieve muscle‐specific knockdown of *Tim* gene expression. By assessing aging‐related phenotypes and investigating the underlying mechanisms of muscle aging, we aimed to elucidate the role of muscle *Tim* in systemic aging, as well as in skeletal and cardiac muscle aging (Figure 2A). Our results showed that muscle‐specific knockdown of *Tim* had no significant effects on 24‐h activity, 12‐h daytime activity, 12‐h nighttime activity, climbing speed, heart rate, stroke volume, or time to hypoxic cardiac failure in 1‐week‐old young flies (Figure 2B–I, *p* > 0.05). In 5‐week‐old aged flies, however, although muscle *Tim* RNAi did not significantly affect 24‐h or daytime activity (Figure 2J,K, *p* > 0.05), it led to a significant increase in nighttime activity (Figure 2L,M, *p* < 0.05 or *p* < 0.01), a marked decline in climbing speed (Figure 2N, *p* < 0.05 or *p* < 0.01), a significant elevation in heart rate (Figure 2O, *p* < 0.05 or *p* < 0.01), and significant reductions in stroke volume (Figure 2P, *p* < 0.05 or *p* < 0.01), time to hypoxic cardiac failure (Figure 2Q, *p* < 0.05 or *p* < 0.01), and lifespan (Figure 2R,S, *p* < 0.05 or *p* < 0.01).

**FIGURE 3:**
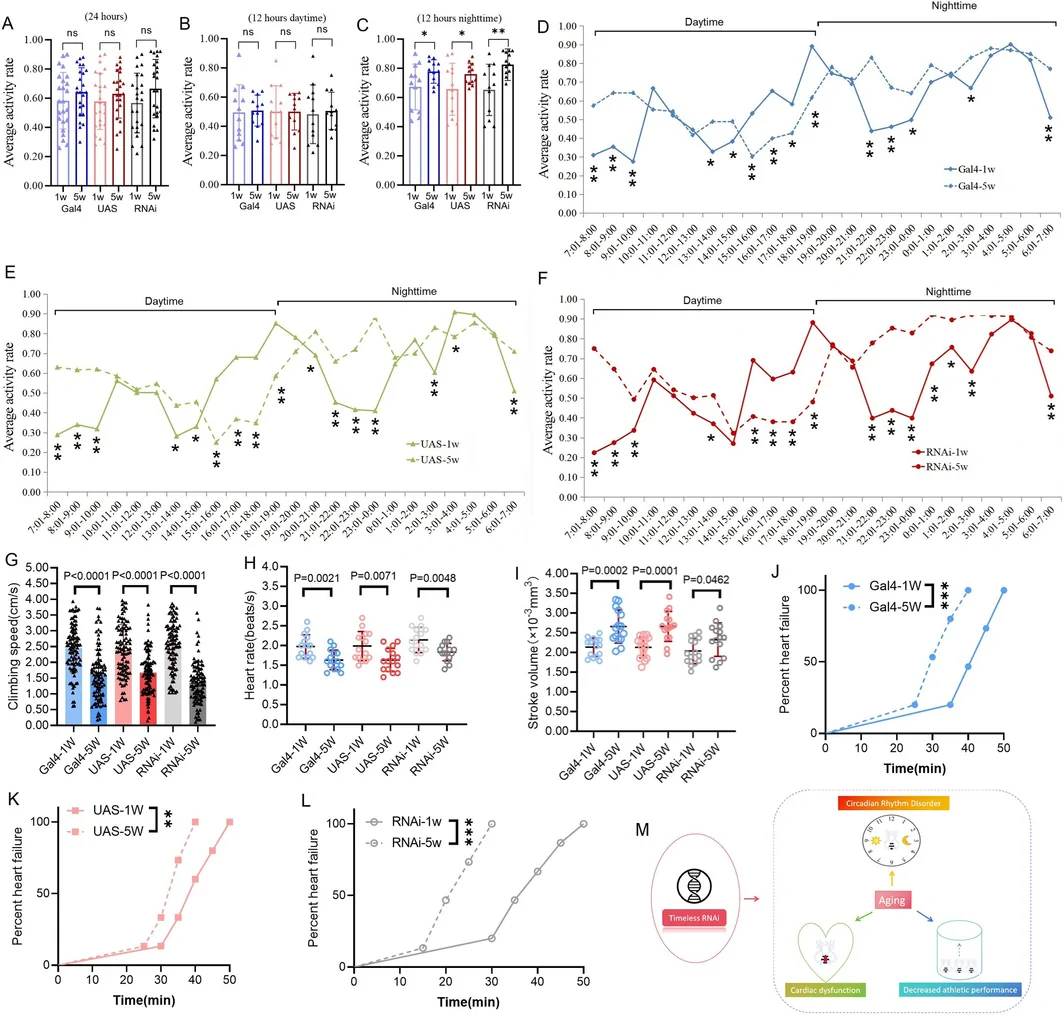
Effects of aging on locomotor activity rhythms, climbing speed, and cardiac function in RNAi flies. (A) Comparison of average 24‐h locomotor activity between 1‐ and 5‐week‐old RNAi flies. (B) Comparison of average 12‐h daytime locomotor activity between 1‐ and 5‐week‐old RNAi flies. (C) Comparison of average 12‐h nighttime locomotor activity between 1‐ and 5‐week‐old RNAi flies. (D) Comparative analysis of 24‐h average locomotor activity profiles between 1‐ and 5‐week‐old GAL4 flies. (E) Comparative analysis of 24‐h average locomotor activity profiles between 1‐ and 5‐week‐old UAS flies. (F) Comparative analysis of 24‐h average locomotor activity profiles between 1‐ and 5‐week‐old RNAi flies. Regardless of whether it is GAL4 flies, UAS flies, or RNAi flies, aging significantly increases their nighttime activity rate, suggesting that aging may lead to increased fragmentation of nighttime sleep in *Drosophila*. However, RNAi may exacerbate this aging‐related nighttime sleep fragmentation. (G) Comparison of climbing speed between 1‐ and 5‐week‐old RNAi flies. (H) Comparison of heart rate between 1‐ and 5‐week‐old RNAi flies. (I) Comparison of cardiac stroke volume between 1‐ and 5‐week‐old RNAi flies. (J) Comparison of time to hypoxic heart failure between 1‐ and 5‐week‐old Gal4 flies. (K) Comparison of time to hypoxic heart failure between 1‐ and 5‐week‐old UAS flies. (L) Comparison of time to hypoxic heart failure between 1‐ and 5‐week‐old RNAi flies. (M) Muscle‐specific *Timeless* gene RNAi accelerated the deterioration of aging‐related behavioral phenotypes. Independent‐sample t‐tests were used to compare the 1‐ and 5‐week‐old flies. The log‐rank test was used to calculate *p*‐values for time to hypoxic heart failure comparisons. Data are presented as means ± standard error of the mean (SEM). A significance threshold set at *p* < 0.05, and ns means no significant difference. *p < 0.05; **p < 0.01; ns means no significant difference.

In addition, the results showed that in flies with different genetic backgrounds (UAS, Gal4, RNAi), there were no significant differences in 24‐h activity or 12‐h daytime activity between 5‐week‐old aged flies and 1‐week‐old young flies (Figure 3A,B, *p* > 0.05). However, the 12‐h nighttime activity of 5‐week‐old aged flies was significantly increased compared to that of 1‐week‐old young flies (Figure 3C–F, *p* < 0.05 or *p* < 0.01). Furthermore, compared to 1‐week‐old young flies, the 5‐week‐old aged flies exhibited significantly reduced climbing speed and heart rate (Figure 3G,H, *p* < 0.05 or *p* < 0.01), a significantly increased stroke volume (Figure 3I, *p* < 0.05 or *p* < 0.01), and a significantly shortened time to hypoxic cardiac failure (Figure 3K,L, *p* < 0.05 or *p* < 0.01). These results suggest that muscle‐specific *Timeless* gene RNAi accelerated the deterioration of aging‐related behavioral phenotypes (Figure 3M).

**FIGURE 4:**
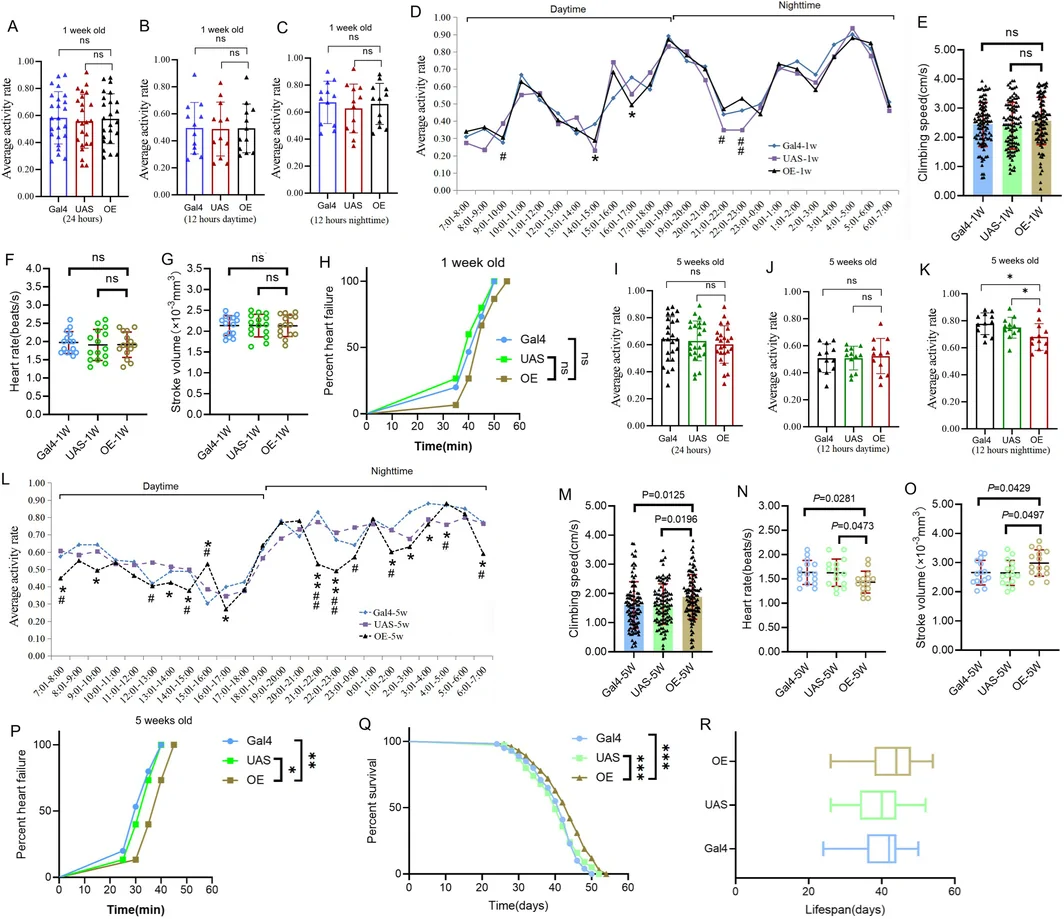
Effects of muscle‐specific *Tim* overexpression (OE) on aging‐related phenotypes in *Drosophila*. (A) Average 24‐h locomotor activity of 1‐week‐old OE flies. (B) Average 12‐h daytime locomotor activity of 1‐week‐old OE flies. (C) Average 12‐h nighttime locomotor activity of 1‐week‐old OE flies. (D) Locomotor activity profile over 24 h of 1‐week‐old OE flies. (E) Climbing speed of 1‐week‐old OE flies. (F) Heart rate of 1‐week‐old OE flies. (G) Stroke volume of 1‐week‐old OE flies. (H) Time to hypoxic heart failure in 1‐week‐old OE flies. (I) Average 24‐h locomotor activity of 5‐week‐old OE flies. (J) Average 12‐h daytime locomotor activity of 5‐week‐old OE flies. (K) Average 12‐h nighttime locomotor activity of 5‐week‐old OE flies. (L) Locomotor activity profile over 24 h of 5‐week‐old OE flies. The nighttime locomotor activity of 5‐week‐old OE flies was significantly lower than that of UAS and GAL4 flies, suggesting that OE may alleviate the fragmentation of nighttime sleep in aged flies. (M) Climbing speed of 5‐week‐old OE flies. (N) Heart rate of 5‐week‐old OE flies. (O) Stroke volume of 5‐week‐old OE flies. (P) Time to hypoxic heart failure in 5‐week‐old OE flies. (Q) Survival curve of OE flies. (R) Average lifespan of OE flies. For comparisons among multiple groups (Gal4, UAS, and OE), one‐way analysis of variance (ANOVA) followed by least significant difference (LSD) post hoc tests was conducted. The log‐rank test was used to calculate *p*‐values for lifespan curve and time to hypoxic heart failure comparisons. Data are presented as means ± standard error of the mean (SEM). *p < 0.05; **p < 0.01; ns means no significant difference.

To further confirm the role of the muscle‐specific *Tim* gene in aging, we generated a *Tim‐UAS‐Overexpression (OE)/Mhc‐Gal4* system in F1‐generation *Drosophila* through genetic crosses to achieve *Tim* overexpression (OE) specifically in muscle tissue. The results showed that muscle‐specific overexpression of *Tim* had no significant effects on the 24‐h activity rate, 12‐h daytime activity rate, 12‐h nighttime activity rate, climbing speed, heart rate, stroke volume, or time to hypoxic cardiac failure in 1‐week‐old young flies (Figure 4A–H, *p* > 0.05). In 5‐week‐old aged flies, however, although muscle‐specific overexpression of *Tim* did not significantly affect their 24‐h or 12‐h daytime activity rates (Figure 4I,J, *p* > 0.05), it significantly reduced their nighttime activity rate (Figure 4K,L, *p* < 0.05 or *p* < 0.01), significantly increased their climbing speed (Figure 4M, *p* < 0.05 or *p* < 0.01), significantly decreased their heart rate (Figure 4N, *p* < 0.05 or *p* < 0.01), and significantly enhanced their stroke volume (Figure 4O, *p* < 0.05 or *p* < 0.01), time to hypoxic cardiac failure (Figure 4P, *p* < 0.05 or *p* < 0.01), and lifespan (Figure 4Q,R, *p* < 0.05 or *p* < 0.01).

**FIGURE 5:**
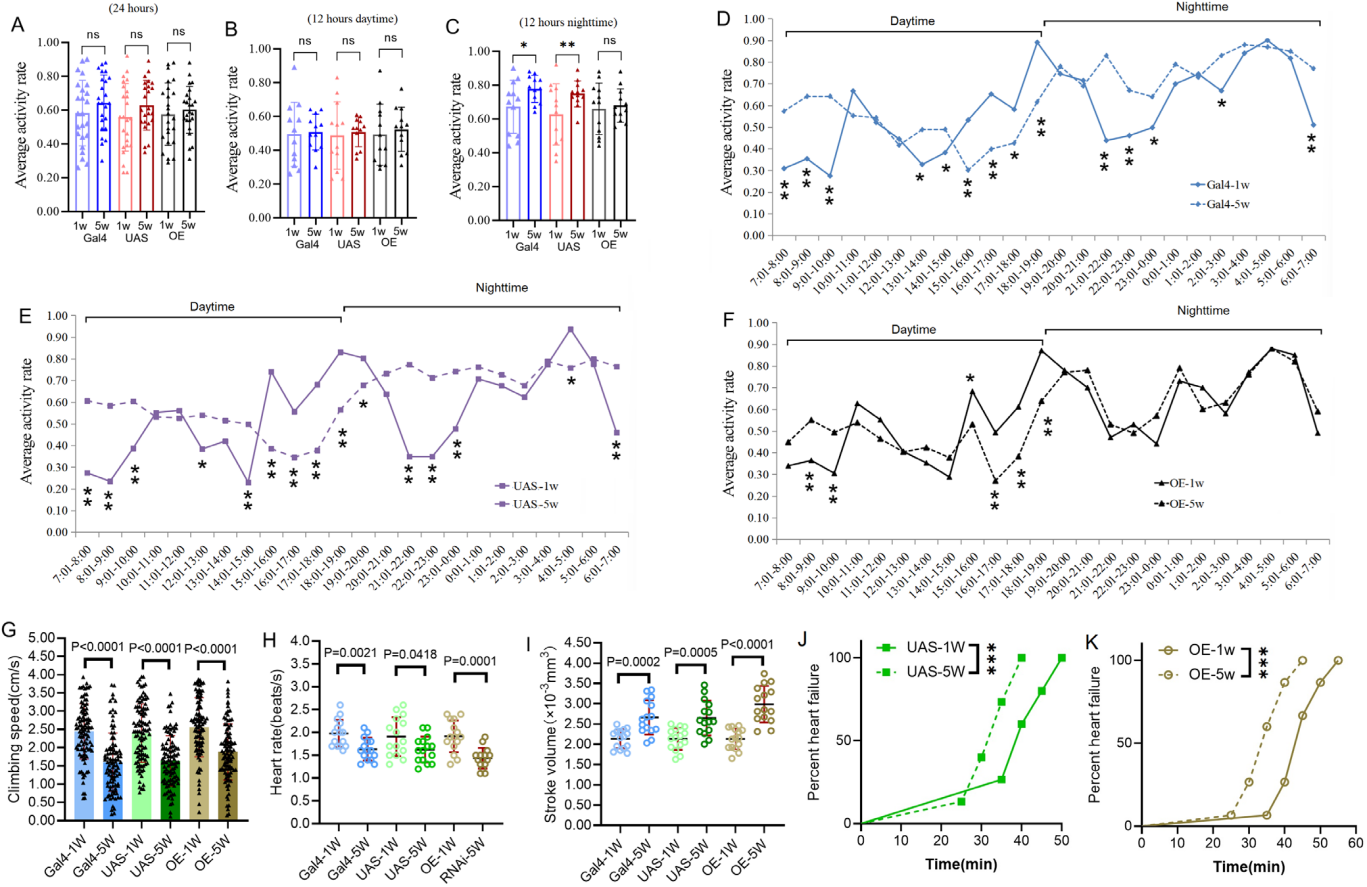
Effects of aging on locomotor activity rhythms, climbing speed, and cardiac function in OE flies. (A) Comparison of average 24‐h locomotor activity between 1‐ and 5‐week‐old OE flies. (B) Comparison of average 12‐h daytime locomotor activity between 1‐ and 5‐week‐old OE flies. (C) Comparison of average 12‐h nighttime locomotor activity between 1‐ and 5‐week‐old OE flies. (D) Comparative analysis of 24‐h average locomotor activity profiles between 1‐ and 5‐week‐old GAL4 flies. (E) Comparative analysis of 24‐h average locomotor activity profiles between 1‐ and 5‐week‐old UAS flies. (F) Comparative analysis of 24‐h average locomotor activity profiles between 1‐ and 5‐week‐old OE flies. Regardless of whether it is GAL4 flies, UAS flies, or RNAi flies, aging significantly increases their nighttime activity rate, suggesting that aging may lead to increased nighttime sleep fragmentation in *Drosophila*. However, this phenomenon was not observed in OE flies, indicating that OE may reduce nighttime sleep fragmentation in aged flies. (G) Comparison of climbing speed between 1‐ and 5‐week‐old OE flies. (H) Comparison of heart rate between 1‐ and 5‐week‐old OE flies. (I) Comparison of cardiac stroke volume between 1‐ and 5‐week‐old RNAi flies. (J) Comparison of time to hypoxic heart failure between 1‐ and 5‐week‐old UAS flies. (K) Comparison of time to hypoxic heart failure between 1‐ and 5‐week‐old OE flies. Independent‐sample t‐tests were used to compare the 1‐ and 5‐week‐old flies. The log‐rank test was used to calculate *p*‐values for time to hypoxic heart failure comparisons. Data are presented as means ± standard error of the mean (SEM). A significance threshold is set at *p* < 0.05, and ns means no significant difference. *p < 0.05; **p < 0.01; ns means no significant difference.

In addition, the results showed that in flies with different genetic backgrounds (UAS, Gal4, OE), there were no significant differences in 24‐h activity or 12‐h daytime activity between 5‐week‐old aged flies and 1‐week‐old young flies (Figure 5A,B, *p* > 0.05). However, the 12‐h nighttime activity of 5‐week‐old aged flies was significantly increased compared to that of 1‐week‐old young flies (Figure 5C–F, *p* < 0.05 or *p* < 0.01). Furthermore, compared to 1‐week‐old young flies, the 5‐week‐old aged flies exhibited significantly reduced climbing speed and heart rate (Figure 5G,H, *p* < 0.05 or *p* < 0.01), a significantly increased stroke volume (Figure 5I, *p* < 0.05 or *p* < 0.01), and a significantly shortened time to hypoxic cardiac failure (Figure 5J,K, *p* < 0.05 or *p* < 0.01). These results suggest that muscle‐specific *Timeless* gene overexpression delayed the deterioration of aging‐related behavioral phenotypes.

### 3.2 Physiological Mechanisms of the Impact of Muscular *Tim* Gene on the Aging of Skeletal Muscle and Heart

Skeletal muscle and cardiac aging are accompanied by a series of changes in intrinsic physiological mechanisms, such as increased oxidative stress, mitochondrial dysfunction, and loss of contractile proteins (Tang et al. 2019; Wang, Gan, et al. 2025; Semel et al. 2025). Although studies have shown that the *Tim* gene is closely associated with cellular rhythms and DNA damage repair (Dheekollu et al. 2013), its impact on the physiological mechanisms of cellular aging requires further investigation.

**FIGURE 6:**
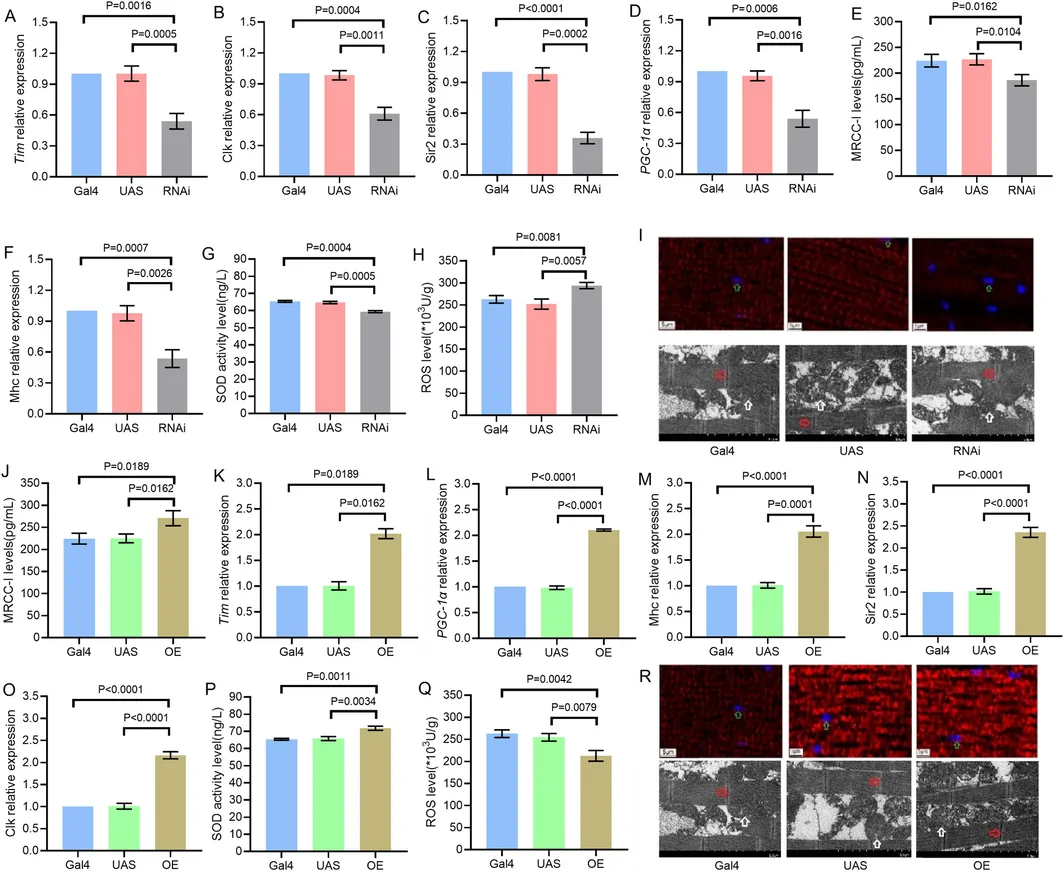
Effects of muscle‐specific Tim RNAi and overexpression (OE) on physiological and biochemical indicators related to *Drosophila* musculature. (A) Relative expression of Tim gene in muscles of RNAi flies. (B) Relative expression of Clk gene in muscles of RNAi flies. (C) Relative expression of Sir2 gene in muscles of RNAi flies. (D) Relative expression of PGC‐1α gene in muscles of RNAi flies. (E) Mitochondrial MRCC‐I protein levels in muscles of RNAi flies. (F) Relative expression of Mhc gene in muscles of RNAi flies. (G) SOD activity levels in muscles of RNAi flies. (H) ROS levels in muscles of RNAi flies. (I) Immunofluorescence of Mhc protein and transmission electron microscopy images of muscles in RNAi flies. (J) Relative expression of Tim gene in muscles of OE flies. (K) Relative expression of Clk gene in muscles of OE flies. (L) Relative expression of Sir2 gene in muscles of OE flies. (M) Relative expression of PGC‐1α gene in muscles of OE flies. (N) Mitochondrial MRCC‐I protein levels in muscles of OE flies. (O) Relative expression of Mhc gene in muscles of OE flies. (P) SOD activity levels in muscles of OE flies. (Q) ROS levels in muscles of OE flies. (R) Immunofluorescence of Mhc protein and transmission electron microscopy images of muscles in OE flies. The immunofluorescence and TEM images of the I‐Gal4 group are identical to those of the R‐Gal4 group, as both were obtained from the same Mhc‐Gal4 driver flies. The first appearance of these images serves to highlight the effect of *Tim* gene knockdown on skeletal muscle, while the second appearance serves to highlight the effect of *Tim* gene overexpression on skeletal muscle. The reuse of these identical images from the same batch of Mhc‐Gal4 flies allows for direct visual comparison of skeletal muscle phenotypes across different experimental conditions, thereby facilitating the assessment of genotype‐specific effects while minimizing inter‐individual variability. For comparisons among multiple groups (Gal4, UAS, and RNAi or OE), one‐way analysis of variance (ANOVA) followed by least significant difference (LSD) post hoc tests was conducted. Data are presented as means ± standard error of the mean (SEM). *p < 0.05; **p < 0.01; ns means no significant difference.

In this study, we first employed real‐time quantitative PCR to detect the mRNA expression levels of the muscular *Tim* gene and others, in order to verify the successful construction of the *Mhc‐Gal4/Tim‐UAS* structure in the F1 generation of *Drosophila*. The results showed that the *Mhc‐Gal4/Tim‐UAS‐RNAi* structure significantly reduced the relative expression of the muscular Tim gene (Figure 6A, *p* < 0.05 or *p* < 0.01). Furthermore, knockdown of the muscular *Tim* gene significantly downregulated the expression of muscle *Clk* gene, *Sir2* gene, and *PGC‐1α* gene, as well as the protein level of MRCC‐I and *Mhc* gene expression, and the activity level of SOD, while significantly increasing the level of muscle ROS (Figure 6B–H, *p* < 0.05 or *p* < 0.01). Results from Mhc protein immunofluorescence histochemistry indicated that knockdown of the muscular *Tim* gene significantly reduced Mhc protein expression. Transmission electron microscopy images of skeletal muscle tissue suggested that knockdown of the muscular Tim gene significantly decreased mitochondrial number and myofibril integrity (Figure 6I).

In contrast, the results showed that the *Mhc‐Gla4/Tim‐UAS‐overexpression* structure significantly up regulated the relative expression of the muscular *Tim* gene (Figure 6J, *p* < 0.05 or *p* < 0.01). Furthermore, overexpression of the muscular *Tim* gene significantly up regulated the expression of muscle *Clk* gene, *Sir2* gene, and *PGC‐1α* gene, as well as the protein level of MRCC‐I and *Mhc* gene expression, and the activity level of SOD, while significantly decreasing the level of muscle ROS (Figure 6K–Q, *p* < 0.05 or *p* < 0.01). Results from Mhc protein immunofluorescence histochemistry indicated that overexpression of the muscular *Tim* gene significantly increased Mhc protein expression. Transmission electron microscopy images of skeletal muscle tissue suggested that overexpression of the muscular *Tim* gene significantly increased mitochondrial number and myofibril integrity (Figure 6R).

It should be noted that the immunofluorescence and TEM images of the Figure 6‐I‐Gal4 group and the Figure 6‐R‐Gal4 group are identical, as both were derived from the same Mhc‐Gal4 driver flies. These images are presented separately in Figure 6I to illustrate the *Tim* knockdown effect and in Figure 6R to illustrate the *Tim* overexpression effect, serving distinct comparative purposes in different genetic contexts. The reuse of these identical images from the same batch of Mhc‐Gal4 flies allows for direct visual comparison of skeletal muscle phenotypes across different experimental conditions, thereby facilitating the assessment of genotype‐specific effects while minimizing inter‐individual variability (Figure 6I,R).

These results indicated that the *Timeless* gene in muscle played an important role in the aging of skeletal muscle, heart, and circadian rhythm, and its mechanism was closely related to its ability to regulate the activity status of the muscle Timeless/Clock pathway and the Timeless/Sir2/PGC‐1α pathway.

### 3.3 Exercise Attenuates Aging in Skeletal Muscle and the Heart via the Upregulation of Muscle *Tim* Gene

Accumulating evidence suggests that regular aerobic exercise helps delay aging‐related phenotypic changes, including improved locomotor activity, enhanced circadian rhythms, and a reduced incidence of cardiovascular diseases in aged individuals (Bruns et al. 2020; Cao et al. 2023; Distefano and Goodpaster 2018). These benefits are associated with the ability of exercise to ameliorate aging‐related physiological changes, such as enhanced antioxidant capacity, reduced oxidative stress, and the preservation of mitochondrial function. However, it remains unclear whether these effects are mechanistically linked to the *Tim* gene in muscle.

**FIGURE 7:**
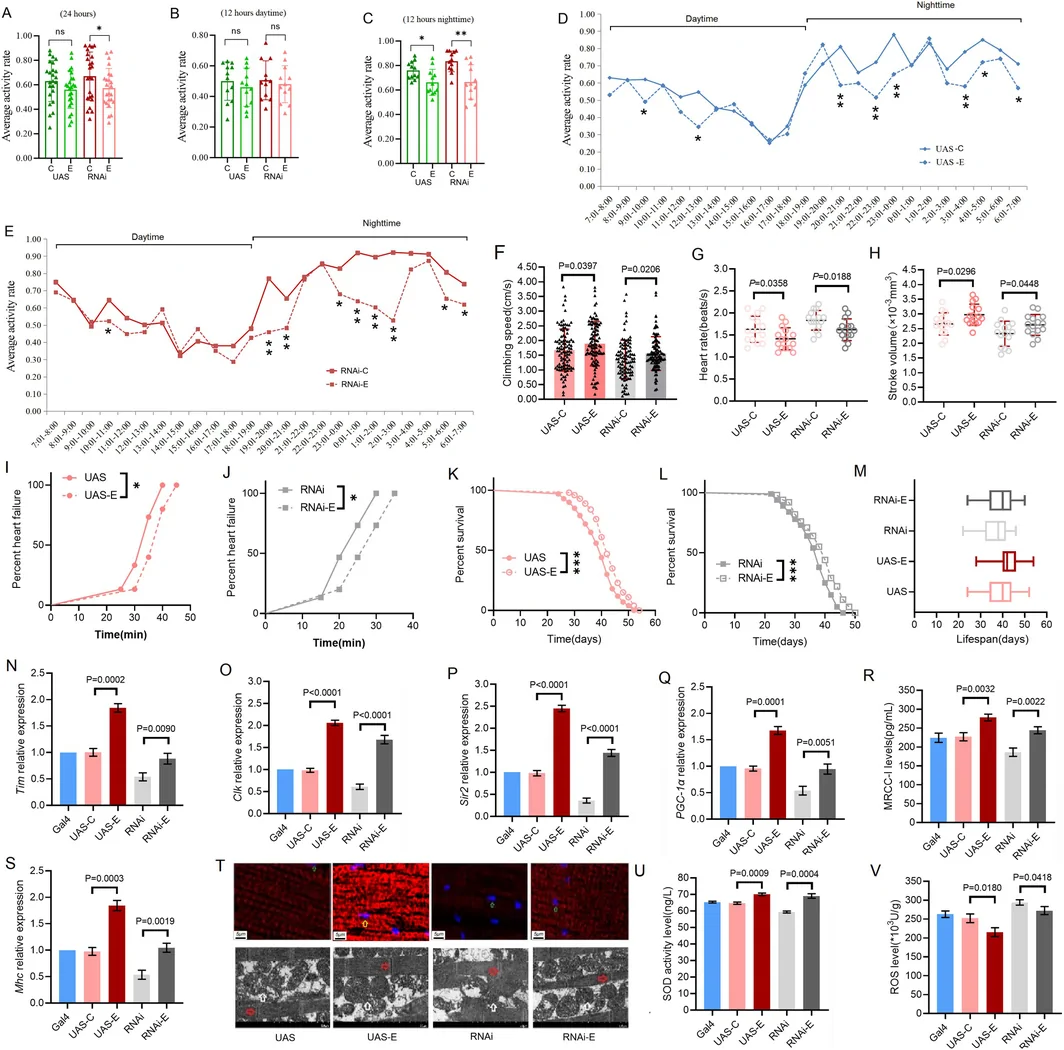
Effects of exercise on muscle‐specific *Tim* RNAi *Drosophila*. (A) Average 24‐h activity rate of flies. (B) Average 12‐h daytime activity rate of flies. (C) Average 12‐h nighttime activity rate of flies. (D) 24‐h average activity rate curve of UAS flies. (E) 24‐h average activity rate curve of RNAi flies. (F) Climbing speed of flies. (G) Heart rate of flies. (H) Stroke volume of flies. (I) Time to hypoxic heart failure in UAS flies. (J) Time to hypoxic heart failure in RNAi flies. (K) Survival curve of UAS flies. (L) Survival curve of RNAi flies. (M) Lifespan of flies. (N) Relative expression of *Tim* gene in muscles. (O) Relative expression of Clk gene in muscles. (P) Relative expression of Sir2 gene in muscles. (Q) Relative expression of PGC‐1α gene in muscles. (R) Mitochondrial MRCC‐I protein levels in muscles. (S) Relative expression of *Mhc* gene in muscles of RNAi flies. (T) Immunofluorescence of Mhc protein and transmission electron microscopy images of muscles in RNAi flies. The immunofluorescence and TEM images of the UAS and RNAi groups in T are identical to those of the UAS and RNAi groups in Figure 6I. In Figure 6I, these images serve to compare the UAS‐RNAi group with the *Tim* RNAi group, highlighting the effect of *Tim* gene knockdown on skeletal muscle, with the primary focus on demonstrating the RNAi effect. In T, the same images are used to compare the UAS group with the UAS‐Exercise group and the RNAi group with the RNAi‐Exercise group, highlighting the effect of exercise on skeletal muscle under conditions of both normal *Tim* expression and *Tim* knockdown, with the primary focus on demonstrating the exercise effect. The reused images are derived from the same cohorts of flies, and the purpose of this reuse is to enable direct visual comparison under different experimental conditions. (U) SOD activity levels in muscles. (V) ROS levels in muscles. Data are presented as means ± standard error of the mean (SEM). A significance threshold is set at *p* < 0.05, and ns means no significant difference. *p < 0.05; **p < 0.01; ns means no significant difference.

The results of this study showed that exercise significantly reduced the 24‐h activity rate and the 12‐h nocturnal activity rate in flies with muscle‐specific *Tim* gene RNA interference (*p* < 0.05 or *p* < 0.01), while no significant effect was observed on their diurnal activity rate (*p* > 0.05) (Figure 7A–E). Moreover, exercise significantly improved the climbing speed of flies with muscle‐specific *Tim* gene RNAi, significantly reduced their heart rate, and markedly enhanced their stroke volume, and it also significantly prolonged the time to heart failure under hypoxia and extended their lifespan (*p* < 0.05 or *p* < 0.01) (Figure 7F–M). Furthermore, exercise significantly increased the mRNA expression of *Tim*, *Clk*, *Sir2*, *PGC‐1α*, and *Mhc*, elevated MRCC‐I protein levels, and transmission electron microscopy images revealed a notable increase in mitochondrial number and a reduction in mitochondrial damage (*p* < 0.05 or *p* < 0.01) (Figure 7N–T). Finally, exercise significantly increased SOD activity and markedly reduced ROS levels in the muscles of muscle‐specific *Tim* RNAi flies (*p* < 0.05 or *p* < 0.01) (Figure 7U,V).

**FIGURE 8:**
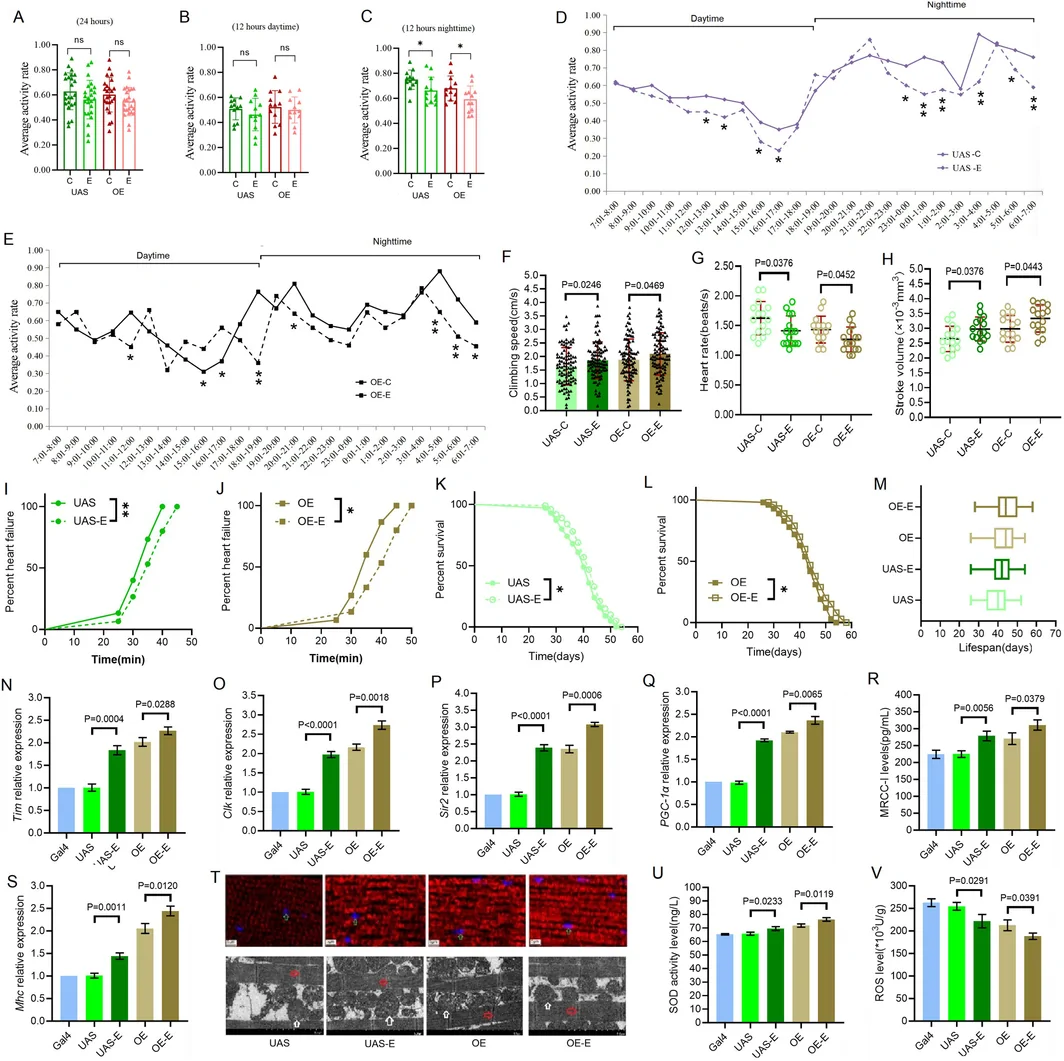
Effects of exercise on muscle‐specific *Tim* overexpression *Drosophila*. (A) Average 24‐h activity rate of flies. (B) Average 12‐h daytime activity rate of flies. (C) Average 12‐h nighttime activity rate of flies. (D) 24‐h average activity rate curve of UAS flies. (E) 24‐h average activity rate curve of overexpression flies. (F) Climbing speed of flies. (G) Heart rate of flies. (H) Stroke volume of flies. (I) Time to hypoxic heart failure in UAS flies. (J) Time to hypoxic heart failure in overexpression flies. (K) Survival curve of UAS flies. (L) Survival curve of overexpression flies. (M) Lifespan of flies. (N) Relative expression of *Tim* gene in muscles. (O) Relative expression of Clk gene in muscles. (P) Relative expression of Sir2 gene in muscles. (Q) Relative expression of PGC‐1α gene in muscles. (R) Mitochondrial MRCC‐I protein levels in muscles. (S) Relative expression of Mhc gene in muscles. (T) Immunofluorescence of Mhc protein and transmission electron microscopy images of muscles in overexpression flies. The immunofluorescence and TEM images of the UAS and OE groups in T are identical to those of the UAS and RNAi groups in Figure 6R. In Figure 6R, these images serve to compare the UAS‐Overexpression group with the *Tim* overexpression group, highlighting the effect of *Tim* gene overexpression on skeletal muscle, with the primary focus on demonstrating the overexpression effect. In T, the same images are used to compare the UAS group with the UAS‐Exercise group and the OE group with the OE‐Exercise group, highlighting the effect of exercise on skeletal muscle under conditions of both normal *Tim* expression and Tim overexpression, with the primary focus on demonstrating the exercise effect. The reused images are derived from the same cohorts of flies, and the purpose of this reuse is to enable direct visual comparison under different experimental conditions. (U) SOD activity levels in muscles. (V) ROS levels in muscles. Data are presented as means ± standard error of the mean (SEM). A significance threshold is set at *p* < 0.05, and ns means no significant difference. *p < 0.05; **p < 0.01; ns means no significant difference.

Similarly, muscle‐specific *Tim* gene overexpression flies, the results of this study showed that exercise also significantly reduced the 24‐h activity rate and the 12‐h nocturnal activity rate in flies with muscle‐specific *Tim* gene overexpression (*p* < 0.05 or *p* < 0.01), while no significant effect was observed on their diurnal activity rate (*p* > 0.05) (Figure 8A–E). Moreover, exercise significantly improved the climbing speed of flies with muscle‐specific *Tim* gene overexpression, significantly reduced their heart rate, and markedly enhanced their stroke volume, and it also significantly prolonged the time to heart failure under hypoxia and extended their lifespan (*p* < 0.05 or *p* < 0.01) (Figure 8F–M). Furthermore, exercise significantly increased the mRNA expression of *Tim*, *Clk*, *Sir2*, *PGC‐1α*, and *Mhc*, elevated MRCC‐I protein levels, and transmission electron microscopy images revealed a notable increase in mitochondrial number and a reduction in mitochondrial damage (*p* < 0.05 or *p* < 0.01) (Figure 8N–T). Finally, exercise significantly increased SOD activity and markedly reduced ROS levels in the muscles of muscle‐specific *Tim* overexpression flies (*p* < 0.05 or *p* < 0.01) (Figure 8U,V).

The immunofluorescence and TEM images of the Figure 7‐T‐UAS and Figure 7‐T‐RNAi groups are identical to those of the Figure 6‐I‐UAS and Figure 6‐I‐RNAi groups, respectively, and the immunofluorescence and TEM images of the Figure 8‐T‐UAS and Figure 8‐T‐OE groups are identical to those of the Figure 6‐R‐UAS and Figure 6‐R‐RNAi groups, respectively. In Figure 6I, the immunofluorescence and TEM images are used to compare the UAS‐RNAi group with the *Tim* RNAi group, highlighting the effect of *Tim* gene knockdown on skeletal muscle, with the primary focus on demonstrating the RNAi effect. In Figure 7T, the immunofluorescence and TEM images are used to compare the UAS group with the UAS‐Exercise group and the RNAi group with the RNAi‐Exercise group, highlighting the effect of exercise on skeletal muscle under conditions of both normal *Tim* expression and *Tim* knockdown, with the primary focus on demonstrating the exercise effect. In Figure 6R, the immunofluorescence and TEM images are used to compare the UAS‐Overexpression group with the *Tim* overexpression group, highlighting the effect of *Tim* gene overexpression on skeletal muscle, with the primary focus on demonstrating the overexpression effect. In Figure 8T, the immunofluorescence and TEM images are used to compare the UAS group with the UAS‐Exercise group and the OE group with the OE‐Exercise group, highlighting the effect of exercise on skeletal muscle under conditions of both normal *Tim* expression and *Tim* overexpression, with the primary focus on demonstrating the exercise effect. The reused images are derived from the same cohorts of flies, and the purpose of this reuse is to enable direct visual comparison under different experimental conditions (Figures 6I,R, 7T, and 8).

These findings suggested that exercise acted as an upstream regulator of the muscle *Timeless* gene, delaying aging‐related deterioration in skeletal muscle, heart, and circadian rhythms by activating the Timeless/Clock and Timeless/Sir2/PGC‐1α pathways.

## 4 Discussion

Aging is an intrinsic process that occurs in nearly all living organisms, characterized by the progressive decline or loss of function across cellular, tissue, and organ systems over time, ultimately leading to the end of life. In humans, the central nervous system is among the most vulnerable to aging, making disruptions in sleep–wake rhythms—such as phase advancement and sleep fragmentation—some of the earliest indicators of the aging process (Brown et al. 2011). These alterations have also been observed in *Drosophila* (Kawasaki et al. 2025), suggesting that age‐related changes in circadian rhythms are evolutionarily conserved.

In mammals, the molecular clock typically exhibits reduced activity during aging, as reflected by decreased amplitude in core clock genes such as Clock, Bmal1, and Per2, while the expression of Per1, Cry1, and Cry2 remains relatively stable. TIM protein directly interacts with the cell cycle checkpoint proteins ATR and CHK1 (Koh et al. 2006). Consequently, the age‐related decline in circadian TIM expression may compromise the function of the CHK1: ATR3 complex in DNA damage checkpoint responses (Gadaleta et al. 2016). In mammals, the *Tim* gene produces two alternatively spliced transcripts, enabling TIM to serve as a molecular link between the cell cycle checkpoint machinery and the circadian clock (Cabral 2025). In *Drosophila*, aging suppresses *Tim* expression, which may contribute to circadian disruption (Umezaki et al. 2012). Collectively, growing evidence implicates *Tim*, a core circadian regulator, in the aging process. However, its specific role in aging skeletal muscle and cardiac tissue remains to be elucidated.

This study employed *Drosophila* genetic crosses to establish a *UAS‐Tim/Mhc‐Gal4* system in F1 progeny, enabling muscle‐specific knockdown and overexpression of the *Tim* gene to investigate its role in circadian rhythm aging, skeletal muscle aging, and cardiac aging. The results showed that muscle‐specific Tim knockdown or overexpression had no significant effect on the average daily locomotor activity in 1‐week‐old young flies. However, in 5‐week‐old aged flies, *Tim* knockdown led to a significant increase in average nighttime activity, whereas *Tim* overexpression significantly reduced nighttime activity. These findings suggest that muscle‐specific *Tim* knockdown accelerates age‐related nighttime sleep fragmentation in *Drosophila*, while *Tim* overexpression helps preserve healthy sleep patterns during aging. These findings are consistent with previously reported studies on circadian rhythms and aging, both in *Drosophila* and mammalian models.

In both mammals and *Drosophila*, skeletal muscle and heart—as vital organs—undergo structural and functional decline with aging, accompanied by an increased incidence of age‐related diseases such as sarcopenia and heart failure. Age‐related sarcopenia is a major cause of reduced locomotor activity and diminished motor capacity in aged individuals, with underlying mechanisms involving loss of contractile proteins in skeletal muscle cells, decreased mitochondrial function, and elevated oxidative stress damage (Fukada and Kajiya 2020; Ji et al. 2025; Scudese et al. 2025). Similarly, age‐associated heart failure is characterized by reduced cardiac contractility, decreased stroke volume and ejection fraction, and increased incidence of arrhythmias, with physiological mechanisms analogous to those observed in skeletal muscle aging (Sato et al. 2025; Liberale et al. 2025). During aging, the reduction in cardiomyocyte number due to apoptosis and necrosis leads to hypertrophy of the remaining cardiomyocytes in an attempt to maintain pump function. However, this hypertrophy increases cardiac stiffness and oxygen consumption (Wang et al. 2024). The myocardium is one of the most oxygen‐consuming tissues in the human body. Hypoxia impairs aerobic oxidation (the most efficient energy production pathway) in cardiomyocytes, forcing a shift toward inefficient anaerobic glycolysis. This results in insufficient energy supply in cardiomyocytes, further deteriorating their systolic and diastolic functions (Lee et al. 2025). Additionally, hypoxia induces mitochondrial dysfunction, generating excessive reactive oxygen species (free radicals) and triggering oxidative stress responses. This damages DNA and proteins within cardiomyocytes while activating inflammatory pathways, thereby accelerating cardiomyocyte death and fibrosis (Klumpe et al. 2016). Hypoxia can also alter the electrophysiological stability of cardiomyocytes, increasing the risk of arrhythmias such as atrial fibrillation, which in turn exacerbates heart failure (Li et al. 2025). Therefore, aging reduces the heart's tolerance to hypoxia and increases the incidence of hypoxic heart failure (Wei et al. 2022). In this study, muscle‐specific knockdown or overexpression of the *Tim* gene had no significant effect on climbing speed or cardiac function in 1‐week‐old young flies. However, in 5‐week‐old aged flies, *Tim* knockdown significantly impaired climbing speed and cardiac function, and shortened the time to heart failure under hypoxic conditions, while *Tim* overexpression produced the opposite effects. These findings suggest that the muscle *Tim* gene plays a critical role in skeletal muscle and cardiac aging.

Sirt1/Sir2, as key NAD^+^‐dependent deacetylases, play a core protective role in skeletal muscle and cardiac aging through multi‐level regulation of mitochondrial function. For instance, in the heart and skeletal muscle, Sirt1/Sir2 activate PGC‐1α via deacetylation, enhancing its transcriptional activity and promoting the expression of NRF1, TFAM, and other factors, thereby facilitating mitochondrial biogenesis and maintaining mitochondrial network stability (Wen et al. 2019; Mu et al. 2014; Ma et al. 2025). Additionally, Sirt1/Sir2 activate the FOXO pathway, upregulating antioxidant enzymes such as SOD2 and catalase to scavenge excess ROS and mitigate oxidative damage in both cardiac and skeletal muscle (Zhang, Zhang, et al. 2025). Conversely, studies have shown that aging is accompanied by a decline in SIRT1/SIRT3 levels, leading to impaired mitochondrial fusion and respiratory function in cardiomyocytes, which subsequently results in defective cardiac contractility (Zhang et al. 2021). SIRT1 deficiency renders the hearts of young mice susceptible to an aging‐like phenotype in response to ischemia/reperfusion injury (Hsu et al. 2017). Although Sirt1/Sir2 are core regulators of skeletal muscle and cardiac aging, the interactive relationship between the *Tim* gene and Sirt1/Sir2 in these tissues remains unclear. This study provides the first evidence in *Drosophila* that muscle *Tim* gene activates the Sir2/PGC‐1α/MRCC‐I pathway and the *Tim‐Clk* pathway, enhances antioxidant capacity (SOD activity), and reduces oxidative stress (ROS), thereby protecting skeletal and cardiac myocytes from age‐related decline and attenuating the loss of contractile proteins during aging.

Accumulating evidence suggests that exercise plays an indispensable and beneficial role in delaying aging. For instance, regular exercise effectively ameliorates age‐related sleep disturbances in the elderly, reduces sleep fragmentation, and enhances circadian rhythm stability in aged individuals (Leise et al. 2013; McHill and Wright 2017). Moreover, both resistance and aerobic exercise attenuate the age‐related loss of skeletal muscle mass, thereby counteracting the decline in physical and locomotor capacity associated with aging (Wang, He, et al. 2025; Bao et al. 2024). Exercise also delays cardiac aging by improving cardiac function and reducing the incidence of age‐related cardiac diseases and heart failure (Kelley et al. 2026). These beneficial effects of exercise on circadian rhythms, skeletal muscle function, and cardiac function are evolutionarily conserved across mammals, humans, and even *Drosophila* (Zheng et al. 2017; Nishimura et al. 2011). However, whether these anti‐aging benefits of exercise are linked to the function of the muscle *Tim* gene remains unclear. The results of this study demonstrate that, regardless of whether the muscle *Tim* gene is knocked down, expressed at normal levels, or overexpressed, exercise improves circadian rhythms (as evidenced by reduced nighttime activity), enhances locomotor capacity and cardiac function, and prolongs the time to hypoxic heart failure in aged *Drosophila*. These findings suggest that exercise may act as an upstream regulator of the muscle *Tim* gene to modulate skeletal muscle and cardiac aging, although the underlying molecular mechanisms remain to be elucidated.

The molecular mechanisms by which exercise delays skeletal muscle and cardiac aging also involve the regulation of Sirt1/Sir2 and its associated pathways. Studies have shown that exercise enhances Sirt1/Sir2 activity by upregulating NAD^+^ levels, thereby activating downstream targets such as PGC‐1α and FOXO. In skeletal muscle, exercise‐induced activation of the Sirt1/PGC‐1α signaling axis promotes mitochondrial biogenesis, enhances oxidative metabolism, and upregulates antioxidant enzyme expression, thereby mitigating age‐related oxidative damage and protein loss (Gao et al. 2024; Zhang, Chen, and wan 2025; Yang et al. 2024). Similarly, in the heart, exercise improves mitochondrial function in cardiomyocytes, enhances contractility, and reduces the incidence of age‐related cardiac lipotoxicity and heart failure through Sirt1‐dependent mechanisms, including the activation of downstream targets such as PGC‐1α and FOXO (Zhang, Chen, and wan 2025; Chen et al. 2023; Wang et al. 2023).

Notably, this study found that the muscle *Tim* gene activates the Sir2/PGC‐1α/MRCC‐I pathway, and exercise also regulates this same pathway, suggesting a potential interaction between *Tim* and Sirt1/Sir2 in mediating the anti‐aging effects of exercise. To further elucidate the relationship between exercise and the muscle *Tim* gene, we examined the relative mRNA expression of Tim in the muscle tissue of aged *Drosophila*. The results showed that exercise significantly upregulated Tim mRNA expression in the muscle tissue of Tim‐knockdown, normal‐expression, and overexpression flies, accompanied by an increase in muscle Clk gene expression. These findings suggest that in aging muscle, exercise acts as an important upstream regulator of the *Tim* gene, positively modulating the muscle *Tim/Clk* pathway, the Sir2/PGC‐1α/MRCC‐I pathway, and antioxidant capacity, thereby reducing oxidative stress damage and contractile protein loss associated with skeletal muscle and cardiac aging, ultimately enhancing tissue function.

Several limitations of this study should be acknowledged. First, although the *Drosophila* model offers significant advantages for genetic manipulation and aging research, the evolutionary divergence between flies and mammals may limit the direct translational applicability of our findings to human biology. Second, our exercise intervention was performed using a mechanical rotational paradigm, which, while well‐established in fly research, may not fully recapitulate the physiological characteristics of voluntary aerobic exercise in higher organisms. Third, our molecular analyses were primarily confined to the mRNA and protein levels of key pathway components; further mechanistic studies—such as tissue‐specific rescue experiments or direct measurements of NAD^+^/NADH ratios—would be valuable to solidify the causal relationships between Tim gene regulation, Sir2/PGC‐1α signaling, and the observed anti‐aging effects. Fourth, we focused exclusively on male flies to avoid confounding effects of the estrous cycle, and therefore the potential sex‐specific differences in the role of muscle Tim in aging remain unexplored. Finally, while our results demonstrate that exercise upregulates muscle *Tim* expression, the precise upstream molecular signals through which exercise exerts this effect (e.g., mechanical, metabolic, or redox‐sensitive pathways) warrant further investigation. Addressing these limitations in future studies will help to further validate and extend our current findings.

## 5 Conclusion

Based on the current findings, the *Timeless* gene in muscle played an important role in the aging of skeletal muscle, heart, and circadian rhythm, and its mechanism was closely related to its ability to regulate the activity status of the muscle Timeless/Clock pathway and the Timeless/Sir2/PGC‐1α pathway. Exercise acted as an upstream regulator of the muscle *Timeless* gene, delaying aging‐related deterioration in skeletal muscle, heart, and circadian rhythms by activating the Timeless/Clock and Timeless/Sir2/PGC‐1α pathways. This study provides potential molecular targets for the prevention and treatment of age‐related diseases affecting skeletal muscle and the heart.

## Author Contributions

Research idea and study design: Deng‐tai Wen. Data acquisition: Deng‐tai Wen, Ying Lin, Jing‐yao Sun. Data Analysis/Interpretation: Ying‐qi Chen, Gang‐bin Sun, Tian‐shuo Yuan, Deyu Shu. Statistical analysis: Deng‐tai Wen. Supervision: Wen‐qi Hou. Each author contributed during manuscript drafting or revision and approved the final version of the manuscript.

## Funding

This work is supported by the College Youth Innovation Team Program of Shandong Province (no. 2023RW057) and the National Natural Science Foundation of China (no. 32000832).

## Conflicts of Interest

The authors declare no conflicts of interest.

## Data Availability

All the generated data and the analysis developed in this study are included in this article.
